# Complete Genome Sequence of Mycobacterium avium subsp. *paratuberculosis* Strain 42-13-1, Isolated in Japan

**DOI:** 10.1128/MRA.00084-21

**Published:** 2021-04-15

**Authors:** Yuichi Ueno, Yohsuke Ogawa, Yuji Takamura, Reiko Nagata, Satoko Kawaji, Yasuyuki Mori

**Affiliations:** a National Institute of Animal Health, National Agriculture and Food Research Organization, Tsukuba, Ibaraki, Japan; b Aichi Prefectural Chuo Livestock Hygiene Service Center, Okazaki, Aichi, Japan; University of Arizona

## Abstract

Here, we report the complete genome sequence of Mycobacterium avium subsp. *paratuberculosis* strain 42-13-1, isolated from cattle presenting with chronic diarrhea caused by Johne’s disease in Japan, which was assembled via long- and short-read hybrid assembly.

## ANNOUNCEMENT

Mycobacterium avium subsp. *paratuberculosis* is the causative agent of Johne’s disease with chronic diarrhea in ruminants. Although M. avium subsp. *paratuberculosis* has been isolated worldwide (1) and genome sequences have been deposited in databases (2), a genome sequence for a Japanese variant was unavailable prior to this report.

Here, we report the complete genome sequence of M. avium subsp. *paratuberculosis* strain 42-13-1, which was isolated from a 3-year-old male Holstein with chronic diarrhea in Ibaraki prefecture (36°03′N, 11°140′E) in 2010. M. avium-specific antibodies were detected in the serum from the cattle using an enzyme-linked immunosorbent assay kit (Johne Screening-Pourquier; Kyoto Biken Laboratories, Japan), and M. avium subsp. *paratuberculosis*-specific DNA was detected via direct quantitative PCR (3) of a fecal sample from which the strain was isolated after a 2-month incubation at 37°C in ambient air on Middlebrook 7H10 agar-based slants completed with the same ingredients as described previously (3). After cloning a single colony, an isolate designated 42-13-1 was determined to be M. avium subsp. *paratuberculosis* via PCR for IS*900* detection (3) and serves as a reference strain for diagnosing Johne’s disease in Japan (4, 5).

For the genomic DNA extraction, 42-13-1 was cultured using the same method as that used for bacterial isolation. Genomic DNA was extracted from a streak of 42-13-1 on the agar slant using a Johne-Pure-Spin kit (FASMAC, Japan) according to the manufacturer’s instructions with the following modifications: 1 mg/ml RNase (Nippongene, Japan) was added to the lysis buffer for cell disruption using MicroSmash MS-100 (Tomy Seiko, Japan) with 8 s of agitation.

Sequencing was performed by Macrogen Japan Corporation using the PacBio RS II platform (Pacific Biosciences, USA) and the NovaSeq 6000 platform (150-bp paired-end reads) (Illumina, USA). For PacBio sequencing, DNA was fragmented to 20 kbp using a g-TUBE (Covaris, USA), and a DNA library was constructed using a SMRTbell template prep kit (Pacific Biosciences). For NovaSeq sequencing, DNA was fragmentated to 350 bp using a Covaris LE220 ultrasonicator (Covaris), and a DNA library was constructed using a TruSeq DNA PCR-free library prep kit (Illumina). The fragment size and concentration were measured using an Agilent 2100 bioanalyzer (Agilent, USA) with a NanoDrop spectrophotometer (Thermo Fisher Scientific, USA).

PacBio sequencing yielded 107,498 preprocessed reads (1.40 Gbp), and Illumina sequencing yielded 39,172,852 preprocessed reads (5.92 Gbp). The mean length and *N*_50_ value of the PacBio subreads were 8,888 bp and 12,078 bp, respectively. The PacBio subreads were assembled using Canu version 1.0.6 (6). Default parameters were used for all software unless otherwise specified. Illumina reads were trimmed using Platanus_trim (http://platanus.bio.titech.ac.jp/pltanus_trim) and reassembled with Canu-assembled contigs using SPAdes version 3.13.1 using “careful” and “trusted-contigs” (7). Accuracy of the scaffold sequence was confirmed by sequence mapping using Illumina reads with the Burrows-Wheeler Aligner (BWA) version 0.6.2 (8) and SAMtools version 0.1.19 (9). Although three indistinct insertions/deletions of 19, 24, and 45 bp were identified by comparison between sequences of the Canu-constructed contig and the hybrid contig using a BLAST search of *in silico* Molecular Cloning Genomics Edition (IMC-GE) software version 7.32 (In Silico Biology, Japan), these were confirmed by PCR and Sanger sequencing. The length of the complete genome of M. avium subsp. *paratuberculosis* strain 42-13-1 was 4,832,738 nucleotides (nt), and the GC content was 69.3%. The average coverages of the PacBio and Illumina reads were 289.7× and 1,167.5×, respectively. Genome annotation was performed with the NCBI Prokaryotic Genome Annotation Pipeline version 5.0 (10) and consisted of 4,321 coding sequences, 1 rRNA, and 47 tRNAs.

### Data availability.

The complete genome sequence of M. avium subsp. *paratuberculosis* strain 42-13-1 is available under accession number CP066812. The raw sequence is available under Sequence Read Archive accession number DRA011321. The BioProject accession number is PRJDB10997, and the BioSample accession number is SAMD00268082.
